# Pseudodislocation of the Shoulder After a Pathologic Scapula Fracture: A Rare Case Report

**DOI:** 10.7759/cureus.110191

**Published:** 2026-06-03

**Authors:** Alexandros Topaloglou, Georgios Antonoglou, Konstantinos Asteriadis, Odysseas Aleanidis, Aristeidis Vrettakos

**Affiliations:** 1 Department of Orthopedic Surgery and Traumatology – Unit for Sport Injuries, General Hospital of Thessaloniki “Agios Pavlos”, Thessaloniki, GRC; 2 Spine Department and Deformities, Interbalkan European Medical Center, Thessaloniki, GRC

**Keywords:** clinical assessment in shoulder, glenoid version, irreducible shoulder dislocation, orthopedic oncology, rare shoulder dislocation, scapula and shoulder trauma, scapula fracture, shoulder injuries, shoulder pathologies, shoulder pseudodislocation

## Abstract

Shoulder pseudodislocation is a rare clinical entity characterized by radiographic malposition of the humeral head relative to the glenoid despite preserved joint articulation. We present the case of a 79-year-old female with a complex oncologic history involving the scapula who presented to the emergency department with acute shoulder pain. Initial radiographs suggested a glenohumeral dislocation; however, given the patient’s complex oncologic history, a closed reduction was deferred in favor of advanced imaging. Subsequent computed tomography (CT) definitively identified the presentation as a pseudodislocation revealing preserved joint articulation despite significant osseous erosion and a pathological fracture of the glenoid and scapular body. Measurement of the glenoid version using the Friedman technique demonstrated a marked deviation of +28.3° anteversion. The patient was managed conservatively with analgesic therapy and immobilization in a neutral position. This case highlights the clinical importance of distinguishing radiographic malposition from true dislocation to avoid unnecessary and potentially harmful procedural interventions, particularly in patients with complex structural bone pathology.

## Introduction

Shoulder dislocation is a frequent reason for emergency department attendance; however, shoulder pseudodislocation is a rare phenomenon that can easily mimic a true dislocation on initial imaging. Characterized by a radiographic malposition of the humeral head relative to the glenoid fossa without loss of joint articulation, it is typically caused by factors that increase intra-articular pressure (e.g., hemarthrosis) or a loss of muscular support. In the oncologic population, bone destruction resulting from metastatic infiltration or radiation-induced changes can further compromise the mechanical integrity of the joint, leading to structural failures. Recognition of this entity is critical, as a misdiagnosis can lead to painful and inappropriate attempts at closed reduction. This case report describes a rare presentation of shoulder pseudodislocation secondary to a pathological scapular fracture in a patient with multi-system oncologic disease. While inferior humeral subluxation is a known clinical phenomenon, its occurrence secondary to a catastrophic structural collapse and pathological fracture of the scapula represents an exceptionally unique mechanism that is scarcely documented in current orthopedic oncology literature.

## Case presentation

History and physical examination 

A 79-year-old bedridden female presented to the emergency department (ED) with a 15-day history of right shoulder pain and a significantly reduced range of motion. The patient had a complex oncologic history: in 2016, she was diagnosed with diffuse large B-cell lymphoma of the left scapula, treated with chemotherapy and radiotherapy. In 2020, she developed a poorly differentiated non-small cell carcinoma of the right scapula, which also required multimodal treatment. Most recently, in 2024, she was diagnosed with non-small cell lung carcinoma. Other comorbidities included chronic polyneuropathy with generalized muscular weakness and a history of tachycardia.

Imaging and initial management 

Initial anteroposterior (AP) (Figure 1) and lateral radiographs (Figure 2) of the right shoulder demonstrated an apparent widening of the glenohumeral joint space and inferior displacement of the humeral head, raising high suspicion for an inferior shoulder dislocation. While the emergency department (ED) team initially suspected a simple dislocation, the patient’s complex history necessitated a more cautious approach.

**Figure 1:**
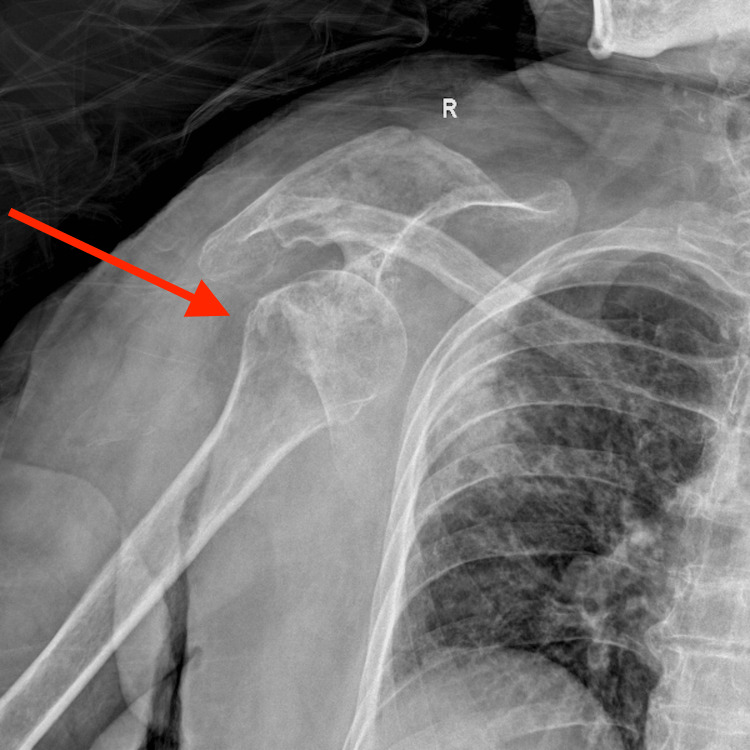
Anteroposterior X-ray of the right shoulder Red arrow: possible inferior shoulder dislocation (difficulty of imaging - bedridden patient)

**Figure 2:**
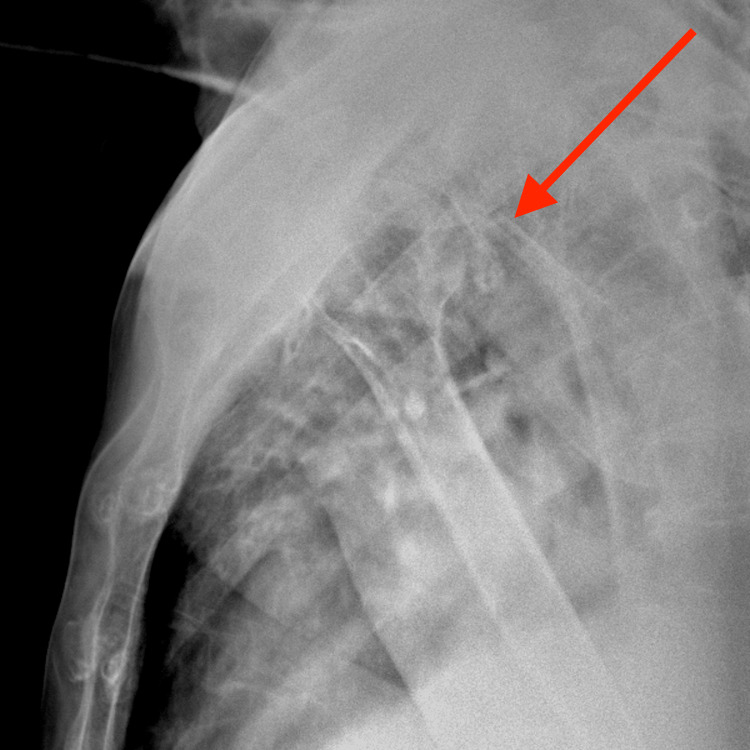
Lateral X-ray of the right shoulder Red arrow: relationship of the humeral head to the scapula (glenoid fossa) (difficulty of imaging - bedridden patient)

It was recognized that a closed reduction should not be attempted on an oncologic patient with a known history of scapular tumors and prior radiotherapy without definitive imaging to rule out structural bone failure. Such maneuvers in the presence of a pathologic fracture could lead to further displacement or unnecessary injury. Based on these findings and the high risk of underlying osseous fragility, an attempt at closed reduction was not performed in the ED, and a diagnostic computed tomography (CT) scan was prioritized to further delineate the anatomy.

Diagnostic refinement 

Given the patient's extensive history of scapular malignancy and the deferral of manual reduction, a CT scan was performed to further delineate the anatomy. The CT imaging revealed that the humeral head maintained its relationship with the remaining glenoid fragment, definitively ruling out a true glenohumeral dislocation.

Instead, the imaging showed extensive osseous erosion and a multi-fragmentary pathological fracture involving the glenoid cavity, the supraspinous fossa, and the area inferior to the scapular spine. Three-dimensional reconstructions (Figures 3-6) further illustrated the catastrophic loss of the lateral scapular border and the structural collapse of the glenoid vault. These findings suggested that the apparent dislocation on the plain radiograph was a false dislocation secondary to structural deformity.

**Figure 3:**
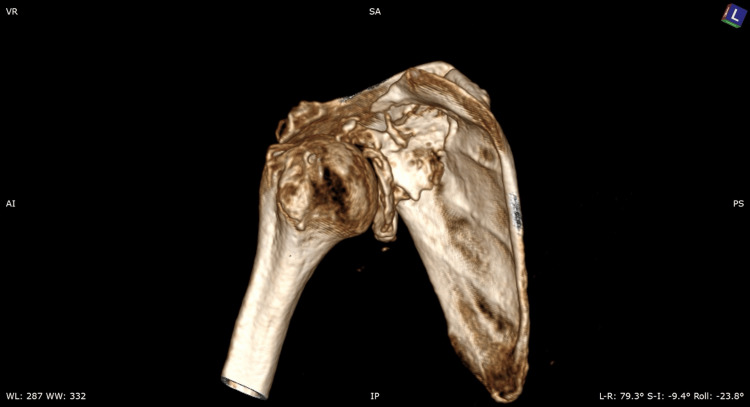
3D CT reconstruction of the right shoulder Humeral head maintained its relationship with the remaining glenoid fragment - ruling out true glenohumeral dislocation

**Figure 4:**
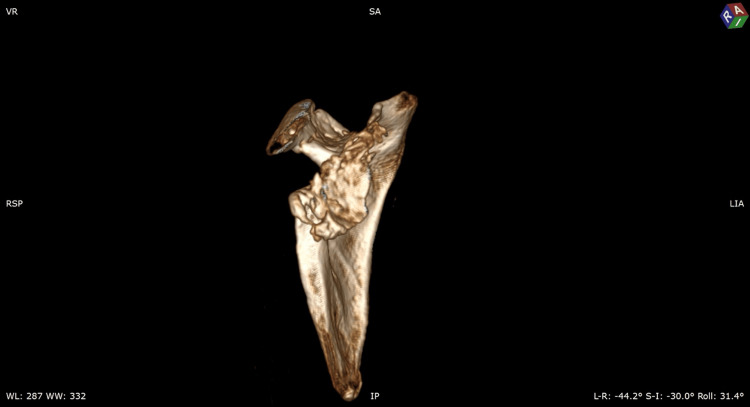
3D CT reconstruction of the right scapula (lateral view)

**Figure 5:**
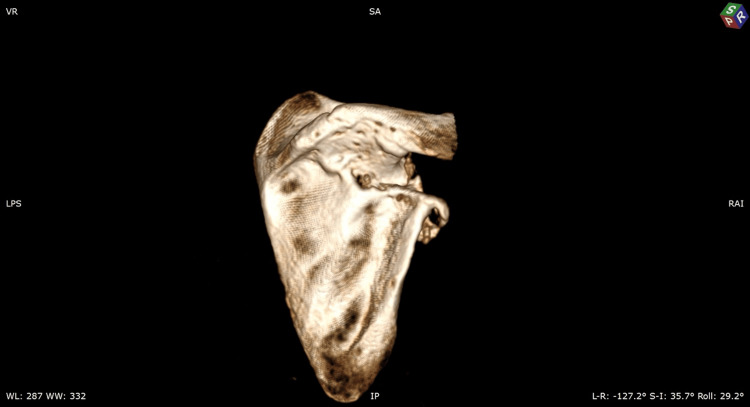
3D CT reconstruction of the right scapula (posterior view)

**Figure 6:**
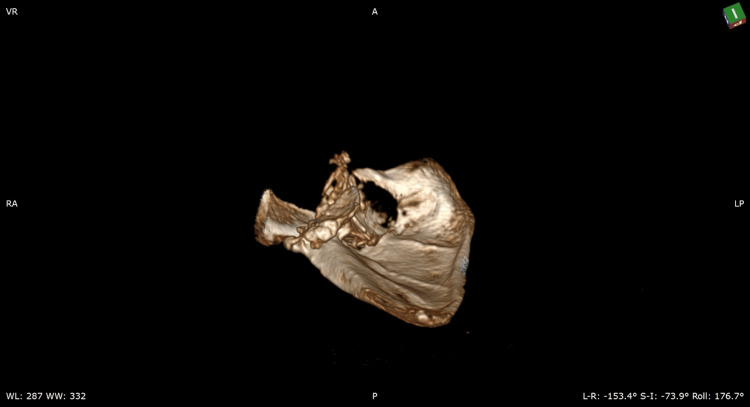
3D CT reconstruction of the right scapula (inferior view)

Treatment and outcome 

Following a multidisciplinary discussion and consultation with the patient and her family, a conservative management plan was adopted. Surgical reconstruction was deemed inappropriate due to the patient’s significant comorbidities and poor functional baseline. The right upper limb was immobilized in a sling in a neutral position at approximately 90° of elbow flexion. Pain was managed effectively with a multimodal analgesic regimen.

## Discussion

Biomechanical analysis and the Friedman technique 

The anatomy of the glenoid, and specifically its anteversion or retroversion, is a critical factor in determining the apparent radiographic relationship of the humeral head to the scapula. Various studies have investigated glenoid version measurements, most commonly using either 2D CT scans or 3D reconstructions, applying methods such as the Friedman technique or the scapular body method [1]. Renna et al. [2] retrospectively analyzed 65 CT scans performed for arthroplasty planning and reported a mean glenoid version of -3.48° (SD 8.7°) using 2D CT.

In the present case, the presence of a scapular fracture complicated the use of the scapular body method for accurate version calculation, as the anatomical landmarks were disrupted. For this reason, the Friedman method was selected on a 2D CT scan. To determine the version, a line was drawn between the anterior and posterior margins of the glenoid (blue). The transverse axis of the scapula was determined by a line connecting the midpoint of the glenoid fossa to the medial end of the scapula, and a perpendicular line to this axis was defined as the neutral version line (red). The glenoid version angle was then calculated relative to this neutral line. In our patient, the measured angle was +28.3° of anteversion (yellow) [1,2], a marked deviation from normal values which likely contributed to the misleading radiographic appearance of a dislocation (Figure 7). 

**Figure 7:**
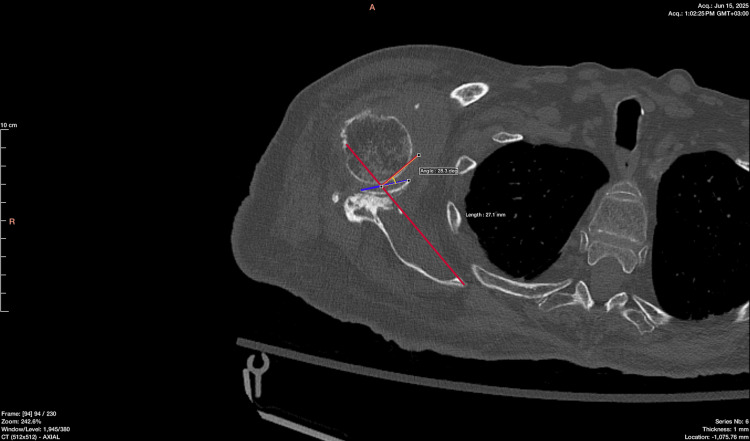
CT scan of the right shoulder, calculating glenoid version using Friedman technique Blue line: drawn between the anterior and posterior margins of the glenoid Red lines: connecting the midpoint of the glenoid fossa to the medial end of the scapula (transverse axis of the scapula was determined), and the perpendicular line to this axis was defined as the neutral version Yellow line: glenoid version angle calculated relative to neutral version, measured angle +28.3° of anteversion

Pathophysiology of shoulder pseudodislocation 

Shoulder pseudodislocation refers to conditions where, despite preserved articulation between the humeral head and the glenoid cavity, the clinical or radiographic presentation strongly mimics a true shoulder dislocation [3]. This is a rare clinical entity, documented primarily through isolated case reports and small case series [4,5]. While increased intra-articular pressure - typically from hemarthrosis or septic arthritis - is the most widely recognized etiology, alternate causes include partial capsular detachment, reflex inhibition of the shoulder musculature, and neurologic deficits secondary to brachial plexus injury [6]. Furthermore, massive inflammatory effusions associated with acute calcific tendinitis and bursitis have recently been shown to produce this deceptive radiographic appearance [3].

In our patient, however, the pseudodislocation was driven by a distinct and formidable mechanism: severe structural and oncologic destruction of the scapula and glenohumeral articulation. 

Diagnostic necessity of computed tomography 

In the clinical management of suspected shoulder instability, CT serves as the definitive diagnostic modality to resolve the ambiguity of conventional radiography. While plain films often present a deceptive image of inferior subluxation, the multiplanar capabilities of CT allow for the precise confirmation of maintained anatomical articulation between the humeral head and the glenoid fossa. This distinction is critical to prevent inappropriate and potentially injurious manual reduction maneuvers.

Beyond joint positioning, CT is instrumental in identifying the underlying pathology responsible for the "drooping shoulder" appearance. In cases involving malignancy, CT provides a detailed assessment of osseous destruction, such as the lytic and osteoblastic lesions associated with metastatic non-small cell lung carcinoma or primary bone lymphoma. It further enables the characterization of complex structural failures, including pathological fractures of the scapular body and glenoid cavity that may be obscured by overlying soft tissues or subtle on 2D X-rays. 

The impact of oncologic disease and radiotherapy 

The patient's oncologic history was a primary driver of the structural failure observed in the right shoulder. The presence of poorly differentiated non-small cell carcinoma within the scapula initiated a process of significant local osteolysis. In such cases, metastatic cells infiltrate the bone matrix and disrupt the balance of bone remodeling, leading to focal areas of weakness and cortical thinning [7].

This malignancy-induced bone loss was further compromised by the patient’s history of radiotherapy. As documented by Zhang and Keener [8], radiation therapy induces late skeletal changes in approximately 1% to 3% of patients. These changes include radiation osteitis, vascular injury to the bone, and a marked decrease in osteoblastic activity, which leaves the bone "static" and unable to repair micro-fractures. In our patient, the synergistic effect of direct tumor erosion and radiation-induced brittleness culminated in a catastrophic pathologic fracture of the scapular body and glenoid. This structural collapse removed the mechanical support for the humeral head, resulting in the characteristic inferior "drooping" [9] identified on the initial radiographs.

Palliative ethics in orthopedic management 

In the management of terminal oncologic patients with pathological fractures, the decision-making process shifts from restorative surgery to a framework of palliative ethics, where the primary objective is the preservation of dignity and the alleviation of suffering. For a frail, bedridden patient, the ethical imperative is to balance the potential for pain relief against the high risk of "overtreatment" and surgical complications [10]. Clinical factors, such as the low union rates and high risk of hardware failure in irradiated bone, often make surgical stabilization a biologically unfavorable option. Consequently, choosing conservative management through optimized analgesia and simple immobilization represents an ethically sound, shared decision-making approach that prioritizes the patient's quality of life over aggressive structural intervention.

Learning points 

The management of this case provides several critical clinical insights. First, it is imperative to distinguish pseudodislocation from true glenohumeral dislocation in patients with an oncologic history to avoid potentially harmful manual reduction maneuvers. In cases involving complex structural bone failure, computed tomography remains the gold standard for defining distorted anatomy and preventing diagnostic errors. Furthermore, the use of objective measurements, such as the Friedman technique to quantify glenoid version, allows clinicians to accurately assess the degree of scapular collapse. Finally, in frail or bedridden patients, clinicians must carefully balance the risks of surgical intervention against a framework of palliative ethics, prioritizing the patient’s quality of life and comfort through shared decision-making.

## Conclusions

Shoulder pseudodislocation is a rare but significant clinical entity that presents a diagnostic challenge in the acute setting, particularly when it mimics a true dislocation on plain films. This case underscores that clinical or radiological signs of joint malposition in patients with a history of malignancy or radiotherapy should be evaluated with a high index of suspicion for structural bone failure rather than simple instability. The use of advanced imaging, specifically computed tomography, is essential to definitively rule out true dislocation and identify underlying pathology, such as pathological fractures or extensive osseous erosion. Measurement of anatomical parameters like the glenoid version can provide further objective clarity on the degree of structural distortion.

In terminal or frail patients, prioritizing palliative ethics through shared decision-making ensures that management goals focus on comfort and the preservation of quality of life. Recognizing this form of malposition early allows for the avoidance of unnecessary, potentially injurious procedural interventions and the timely initiation of appropriate conservative care. By shifting the focus from restorative surgery to symptom management, clinicians can provide care that is both biologically appropriate and ethically sound.
